# Supplementary material for: Sesquiterpene Lactones with Dual Inhibitory Activity against the Trypanosoma brucei Pteridine Reductase 1 and Dihydrofolate Reductase
Source: Molecules. 2021 Dec 27;27(1):149. doi: 10.3390/molecules27010149 (PMC8747069; doi:10.3390/molecules27010149)
Supplement: Supplementary file 1 [file molecules-27-00149-s001.zip › molecules-1508046-supplementary.pdf]

molecules

Supplementary Materials

Sesquiterpene Lactones with Dual Inhibitory Activity against the *Trypanosoma brucei* Pteridine Reductase 1 and Dihydrofolate Reductase

Katharina Possart [1], Fabian C. Herrmann [1], Joachim Jose [2], Maria P. Costi [3] and Thomas J. Schmidt [1,*]

[1] Institute of Pharmaceutical Biology and Phytochemistry (IPBP), University of Muenster, PharmaCampus, Corrensstrasse 48, D-48149 Muenster, Germany; k_poss01@uni-muenster.de (K.P.); f_herr01@uni-muenster.de (F.C.H.)
[2] Institute of Pharmaceutical and Medicinal Chemistry, University of Muenster, PharmaCampus, Corrensstrasse 48, D-48149 Muenster, Germany; joachim.jose@uni-muenster.de
[3] Department of Life Sciences, University of Modena and Reggio Emilia, Via G. Campi 103, 41125 Modena, Italy; mariapaola.costi@unimore.it
* Correspondence: thomschm@uni-muenster.de; Tel.: +49-251-83-33378

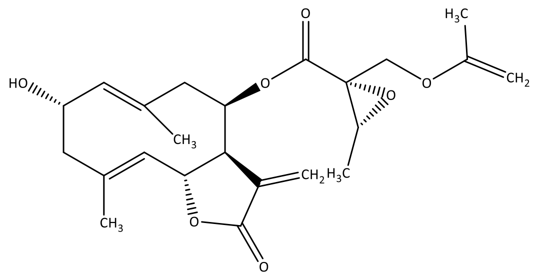

11

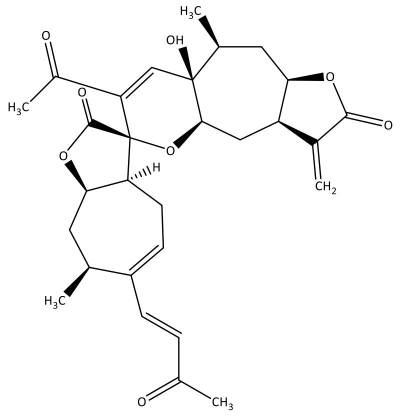

12

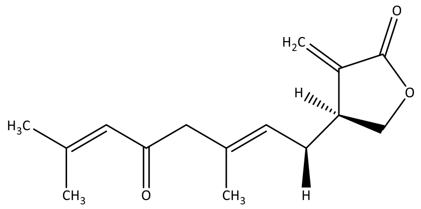

13

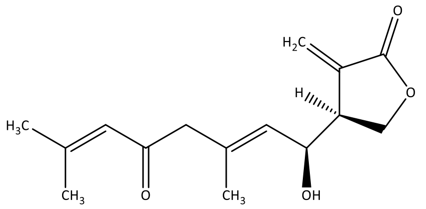

14

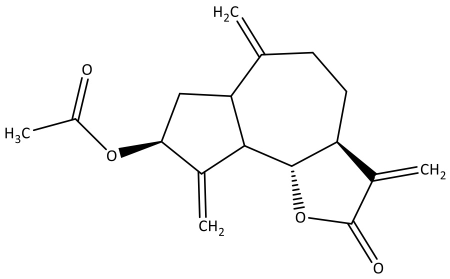

15

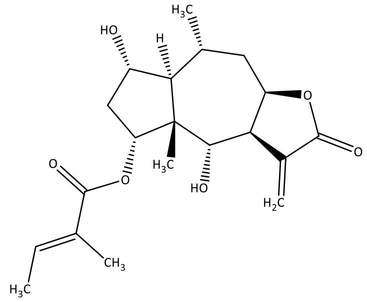

16

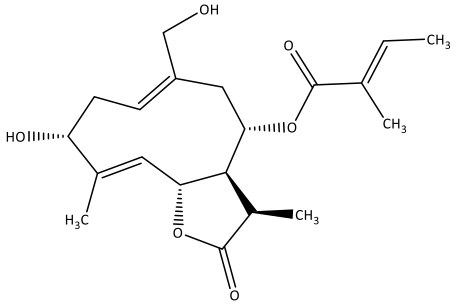

17

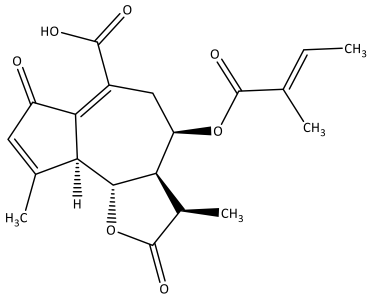

18

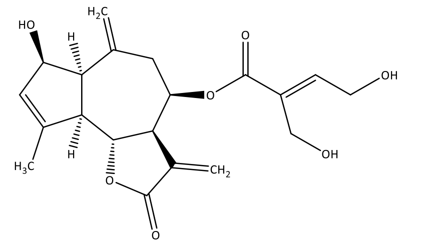

19

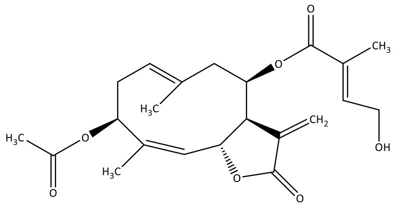

20

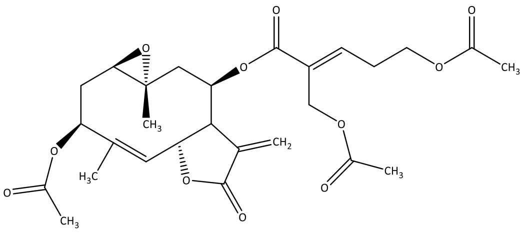

21

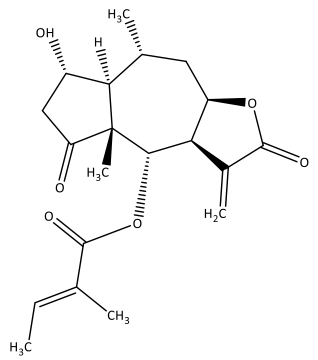

22

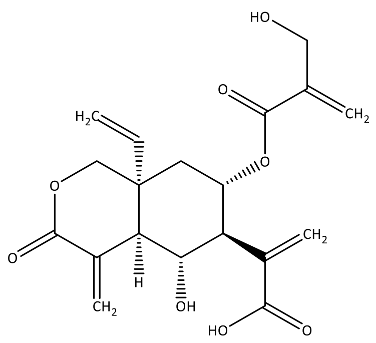

23

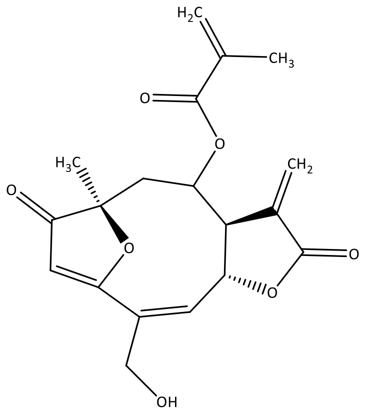

24

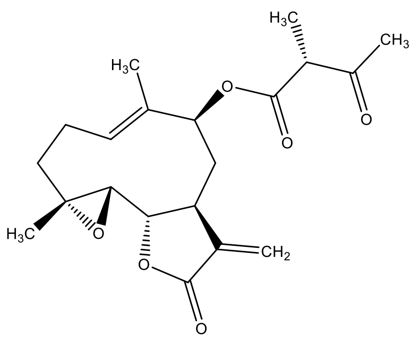

25

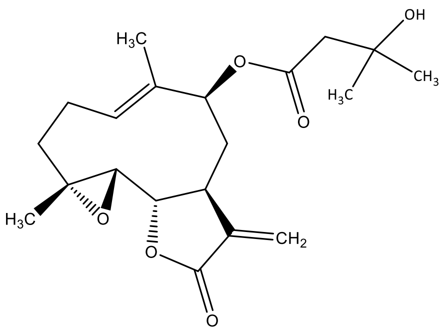

26

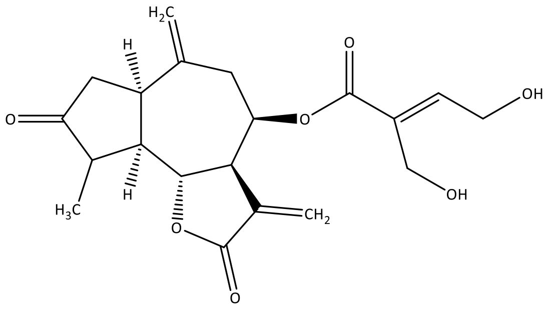

27

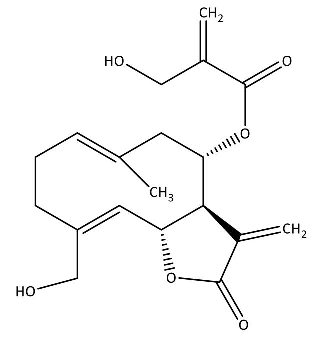

28

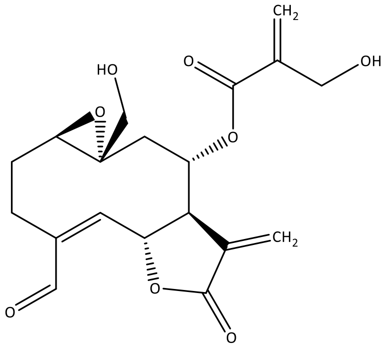

29

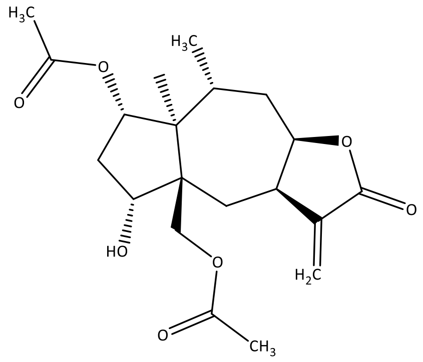

30

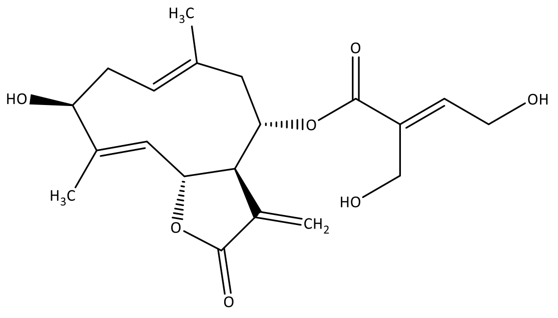

31

Figure S1. Molecular structures of further sesquiterpene lactones identified as *in silico* hits during the pharmacophore-based *in silico* screening but not tested.

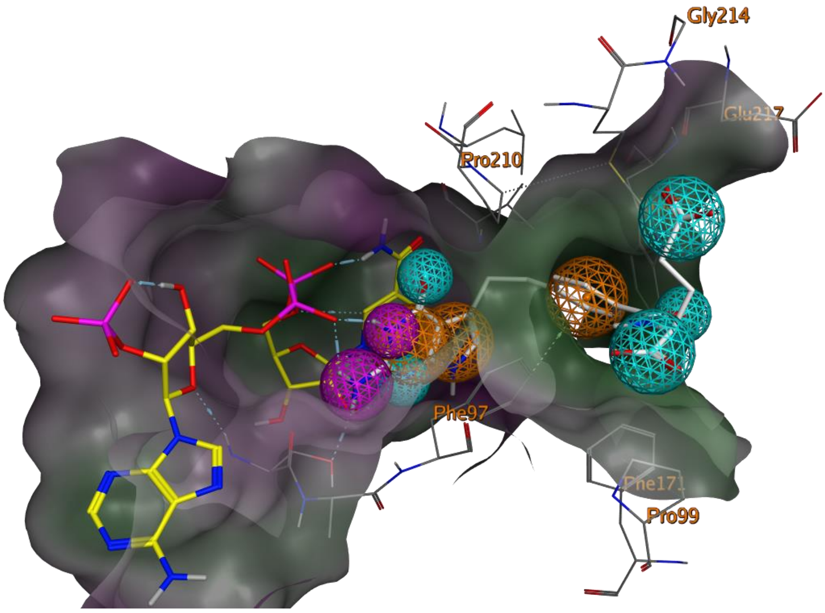

Figure S2. Complex-based pharmacophore hypothesis based on the *Tb*PTR1 binding pocket (ID: „2X9G""). Carbon atoms of the co-crystallized co-substrate NADP in yellow, carbon atoms of the co-crystallized inhibitor pemetrexed in white. The molecular surface is coloured according to lipophilicity with lipophilic areas in green and hydrophilic areas in purple. Potential interactions of the inhibitor are represented by feature spheres: H-bond donors in purple, H-bond acceptors in cyan, aromatic centers in orange. Exclusion spheres are not depicted.

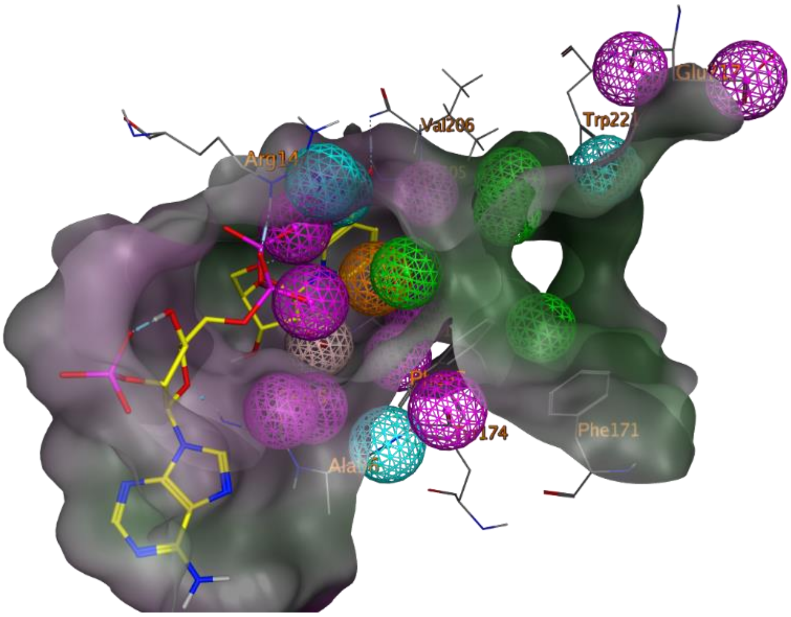

Figure S3. Target-based pharmacophore hypothesis based on the *Tb*PTR1 binding pocket (ID: „2X9G"**)**. Carbon atoms of the co-crystallized co-substrate NADP in yellow, the molecular surface is coloured according to lipophilicity with lipophilic areas in green and hydrophilic areas in purple. Potential interactions of amino acids with an inhibitor are represented by projecting feature spheres: H-bond donors in purple, H-bond acceptors in cyan, ionic interactions in beige, aromatic centers in orange, hydrophobic structures in green. Exclusion spheres are not depicted.

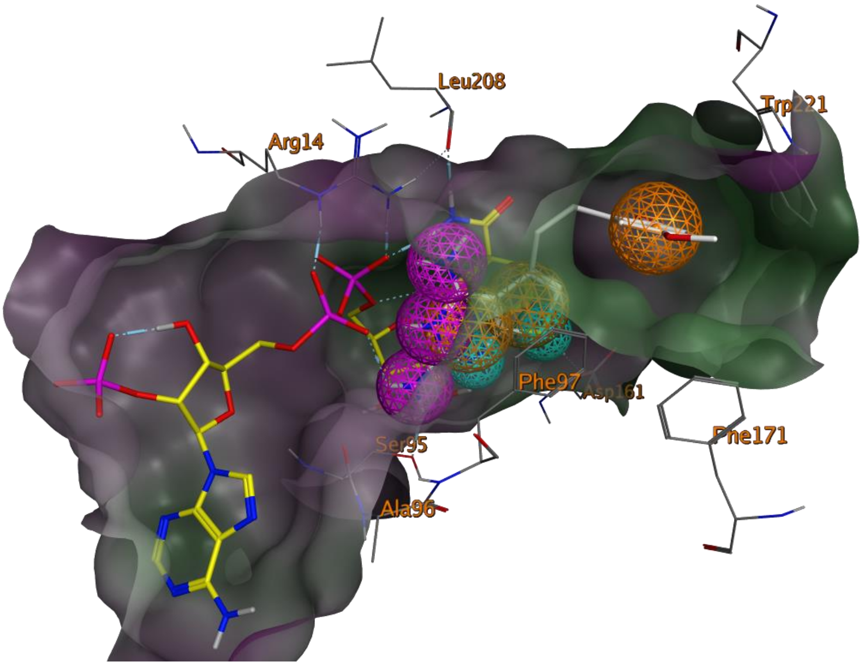

Figure S4. Complex-based pharmacophore hypothesis based on the *Tb*PTR1 binding pocket (ID: „3MCV"). Carbon atoms of the co-crystallized co-substrate NADP in yellow, carbon atoms of the co-crystallized inhibitor PY848 in white. The molecular surface is coloured according to lipophilicity with lipophilic areas in green and hydrophilic areas in purple. Potential interactions of the inhibitor are represented by feature spheres: H-bond donors in purple, H-bond acceptors in cyan, aromatic centers in orange. Exclusion spheres are not depicted.

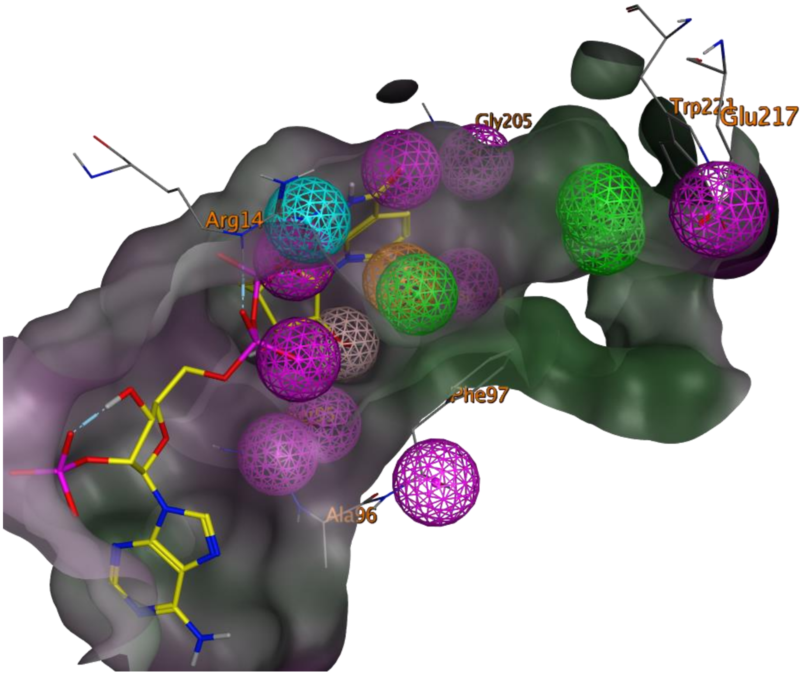

Figure S5. Target-based pharmacophore hypothesis based on the *Tb*PTR1 binding pocket (ID: „3MCV"). Carbon atoms of the co-crystallized co-substrate NADP in yellow, the molecular surface is coloured according to lipophilicity with lipophilic areas in green and hydrophilic areas in purple. Potential interactions of amino acids with an inhibitor are represented by projecting feature spheres: H-bond donors in purple, H-bond acceptors in cyan, ionic interactions in beige, aromatic centers in orange, hydrophobic structures in green. Exclusion spheres are not depicted.

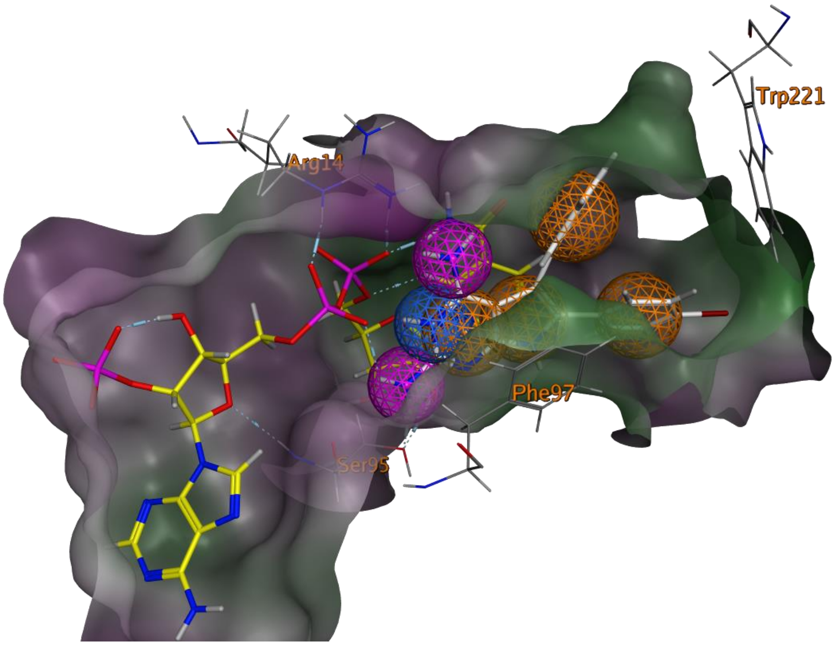

Figure S6. Complex-based pharmacophore hypothesis based on the *Tb*PTR1 binding pocket (ID: „4CMJ“). Carbon atoms of the co-crystallized co-substrate NADP in yellow, carbon atoms of the co-crystallized inhibitor 6 (4 bromophenyl) 5 phenyl 7H-pyrrolo[2,3 d]pyrimidine-2,4-diamine in white. The molecular surface is coloured according to lipophilicity with lipophilic areas in green and hydrophilic areas in purple. Potential interactions of the inhibitor are represented by feature spheres: H-bond donors in purple, cationic interactions in blue, aromatic centres in orange. Exclusion spheres are not depicted.

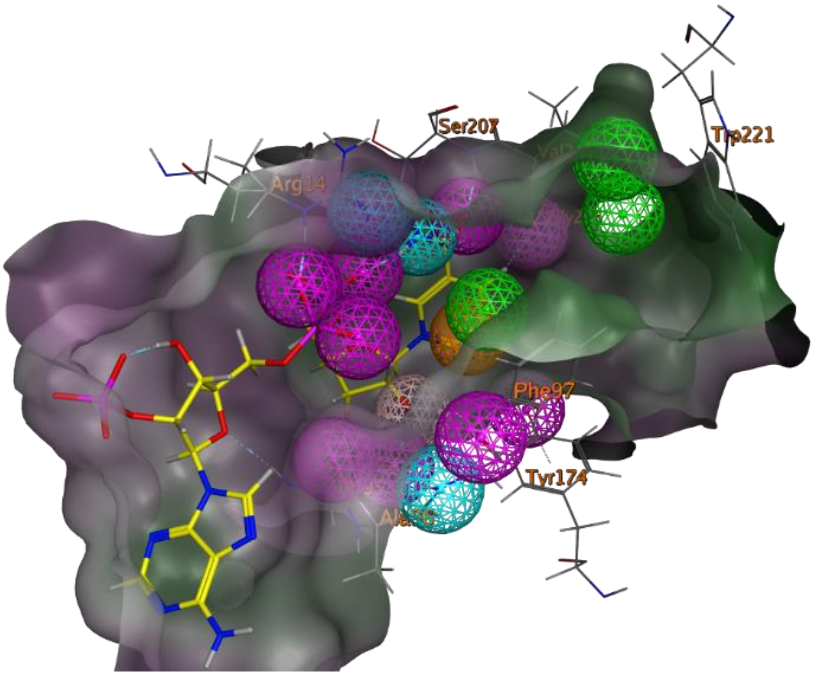

Figure S7. Target-based pharmacophore hypothesis based on the *Tb*PTR1 binding pocket (ID: „4CMJ“). Carbon atoms of the co-crystallized co-substrate NADP in yellow, the molecular surface is coloured according to lipophilicity with lipophilic areas in green and hydrophilic areas in purple. Potential interactions of amino acids with an inhibitor are represented by projecting feature spheres: H-bond donors in purple, H-bond acceptors in cyan, ionic interactions in beige, aromatic centres in orange, hydrophobic structures in green. Exclusion spheres are not depicted.

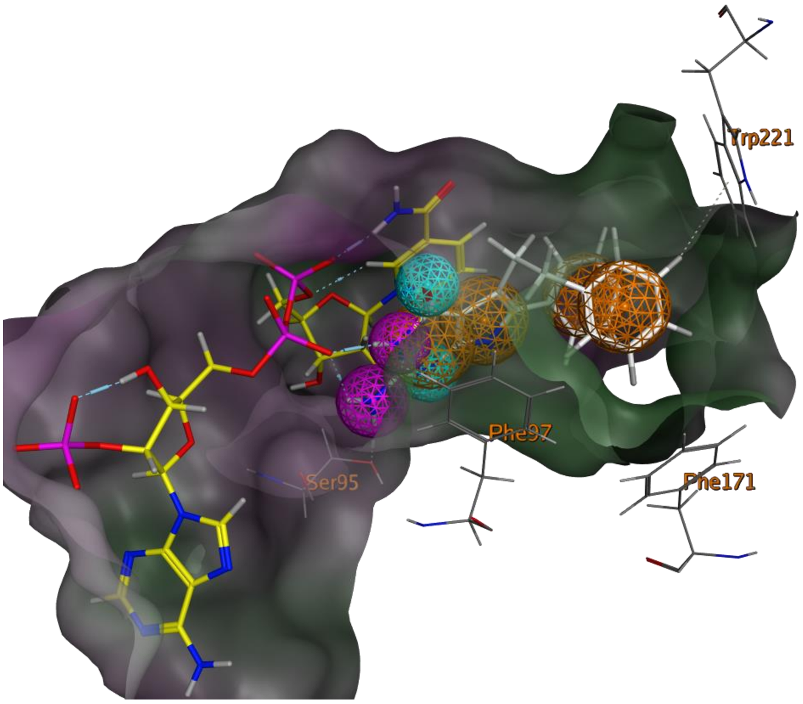

Figure S8. Complex-based pharmacophore hypothesis based on the *Tb*PTR1 binding pocket (ID: „4CMK"). Carbon atoms of the co-crystallized co-substrate NADP in yellow, carbon atoms of the co-crystallized inhibitor 2-amino-5-phenethyl-6-phenyl-3H-pyrrolo[2,3-d]pyrimidine-4(7H)-one in white. The molecular surface is coloured according to lipophilicity with lipophilic areas in green and hydrophilic areas in purple. Potential interactions of the inhibitor are represented by feature spheres: H-bond donors in purple, H-bond acceptors in cyan, aromatic centres in orange. Exclusion spheres are not depicted.

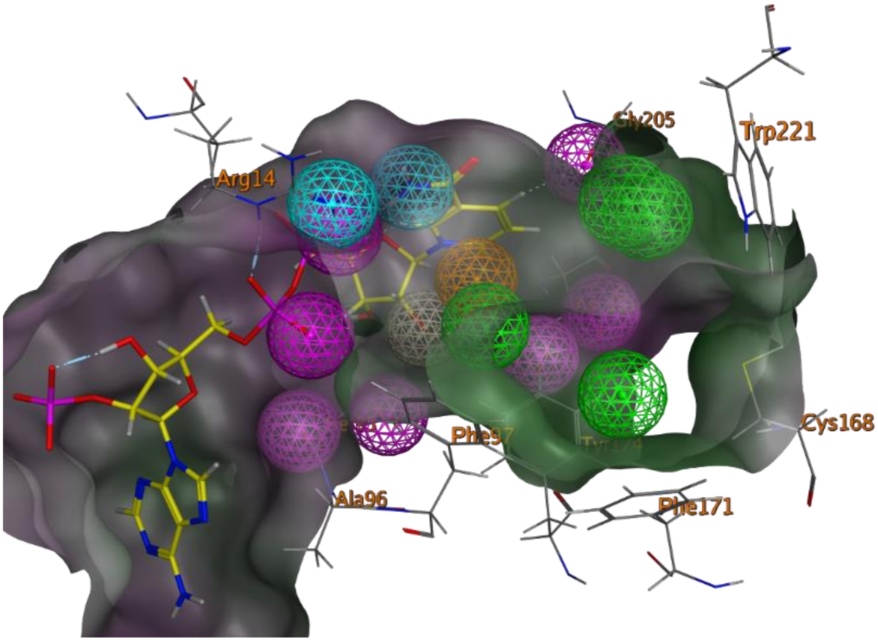

Figure S9. Target-based pharmacophore hypothesis based on the *Tb*PTR1 binding pocket (ID: „4CMK"). Carbon atoms of the co-crystallized co-substrate NADP in yellow, the molecular surface is coloured according to lipophilicity with lipophilic areas in green and hydrophilic areas in purple. Potential interactions of amino acids with an inhibitor are represented by projecting feature spheres: H-bond donors in purple, H-bond acceptors in cyan, ionic interactions in beige, aromatic centres in orange, hydrophobic structures in green. Exclusion spheres are not depicted.

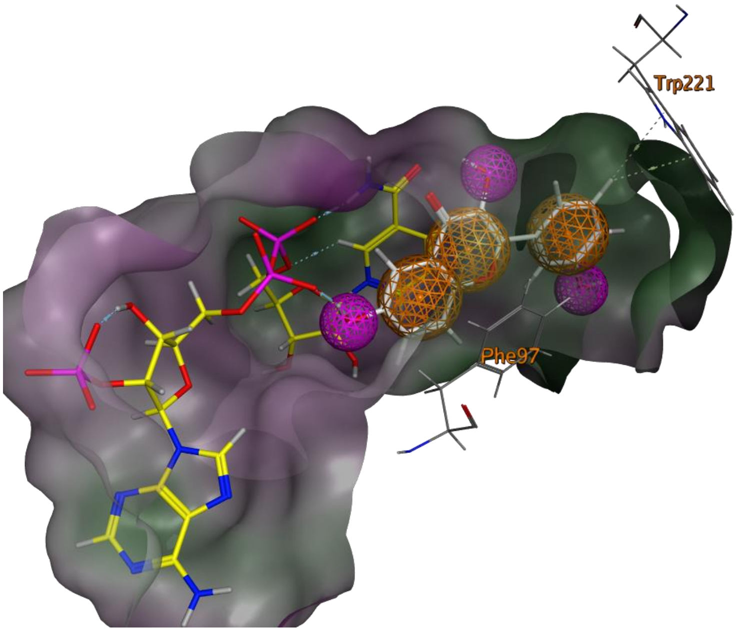

Figure S10. Complex-based pharmacophore hypothesis based on the *Tb*PTR1 binding pocket (ID: „5JDI""). Carbon atoms of the co-crystallized co-substrate NADP in yellow, carbon atoms of the co-crystallized inhibitor 3,6-dihydroxyl-2-(3-hydroxyphenyl)-4H-1-benzopyrane-4-one in white. The molecular surface is coloured according to lipophilicity with lipophilic areas in green and hydrophilic areas in purple. Potential interactions of the inhibitor are represented by feature spheres: H-bond donors in purple, aromatic centres in orange. Exclusion spheres are not depicted.

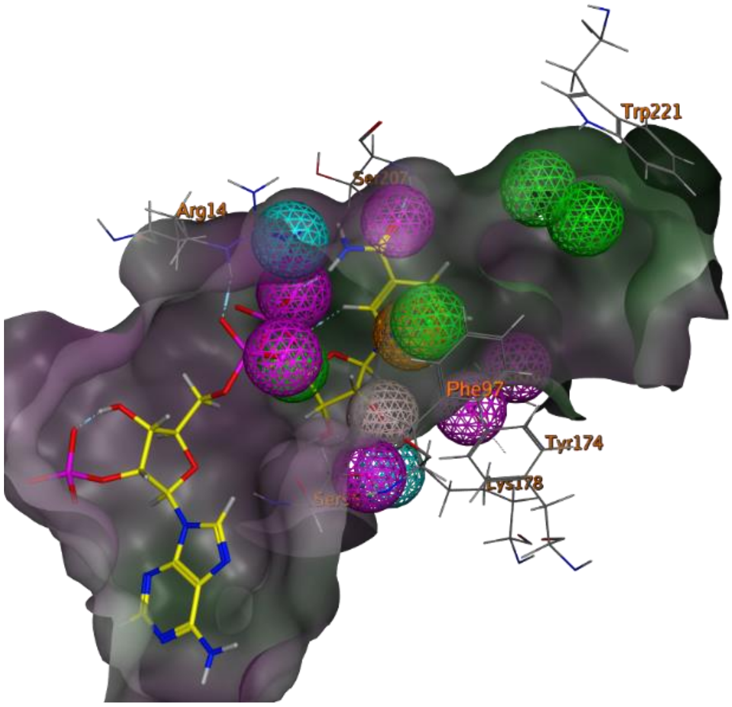

Figure S11. Target-based pharmacophore hypothesis based on the *Tb*PTR1 binding pocket (ID: „5JDI""). Carbon atoms of the co-crystallized co-substrate NADP in yellow, the molecular surface is coloured according to lipophilicity with lipophilic areas in green and hydrophilic areas in purple. Potential interactions of amino acids with an inhibitor are represented by projecting feature spheres: H-bond donors in purple, H-bond acceptors in cyan, ionic interactions in beige, aromatic centres in orange, hydrophobic structures in green. Exclusion spheres are not depicted.

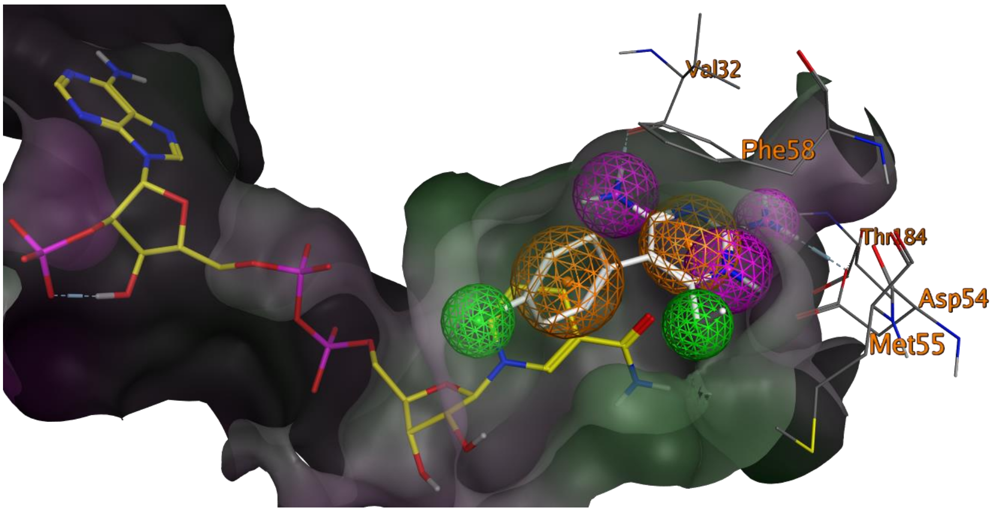

Figure S12. Complex-based pharmacophore hypothesis based on the *Tb*DHFR binding pocket (ID: „3QFX“). Carbon atoms of the co-crystallized co-substrate NADPH in yellow, carbon atoms of the co-crystallized inhibitor pyrimethamine in white. The molecular surface is coloured according to lipophilicity with lipophilic areas in green and hydrophilic areas in purple. Potential interactions of the inhibitor are represented by feature spheres: H-bond donors in purple, aromatic centres in orange, hydrophobic structures in green. Exclusion spheres are not depicted.

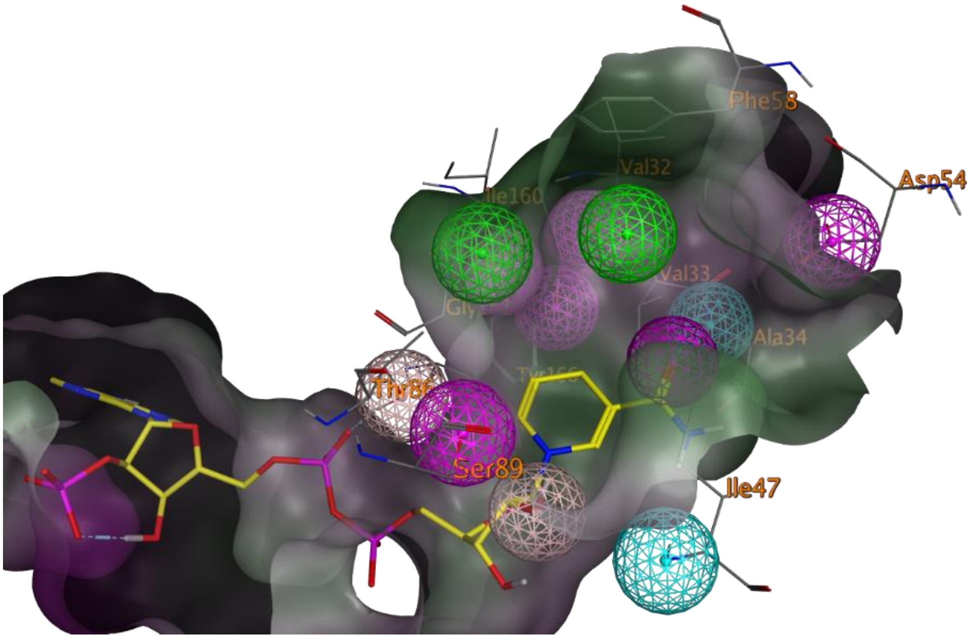

Figure S13. Target-based pharmacophore hypothesis based on the *Tb*DHFR binding pocket (ID: „3QFX“). Carbon atoms of the co-crystallized co-substrate NADPH in yellow, the molecular surface is coloured according to lipophilicity with lipophilic areas in green and hydrophilic areas in purple. Potential interactions of amino acids with an inhibitor are represented by projecting feature spheres: H-bond donors in purple, H-bond acceptors in cyan, ionic interactions in beige, aromatic centres in orange, hydrophobic structures in green. Exclusion spheres are not depicted.

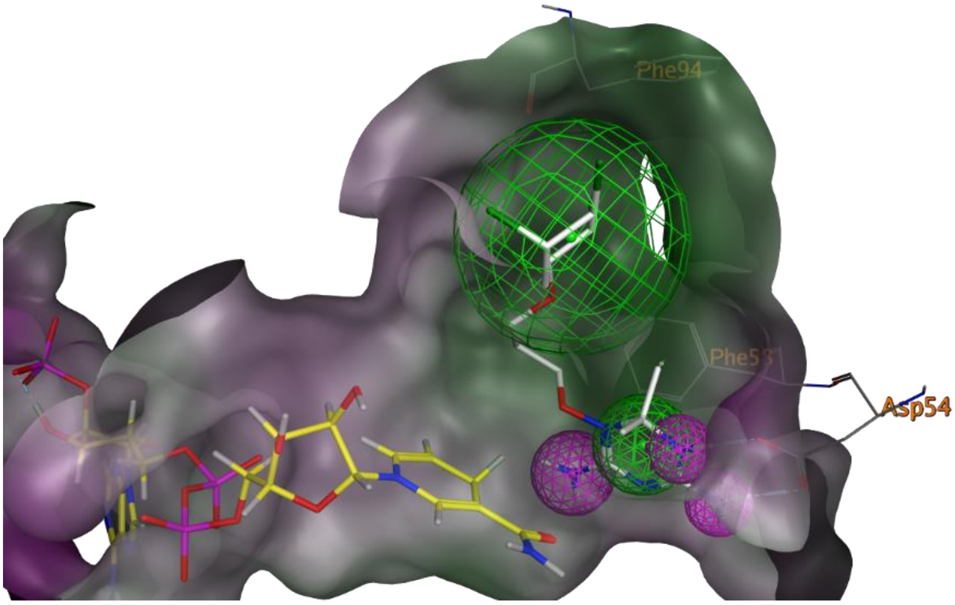

Figure S14. Complex-based pharmacophore hypothesis based on the *Tb*DHFR binding pocket (ID: „3RG9“). Carbon atoms of the co-crystallized co-substrate NADPH in yellow, carbon atoms of the co-crystallized inhibitor WR99210 in white. The molecular surface is coloured according to lipophilicity with lipophilic areas in green and hydrophilic areas in purple. Potential interactions of the inhibitor are represented by feature spheres: H-bond donors in purple, hydrophobic structures in green. Exclusion spheres are not depicted.

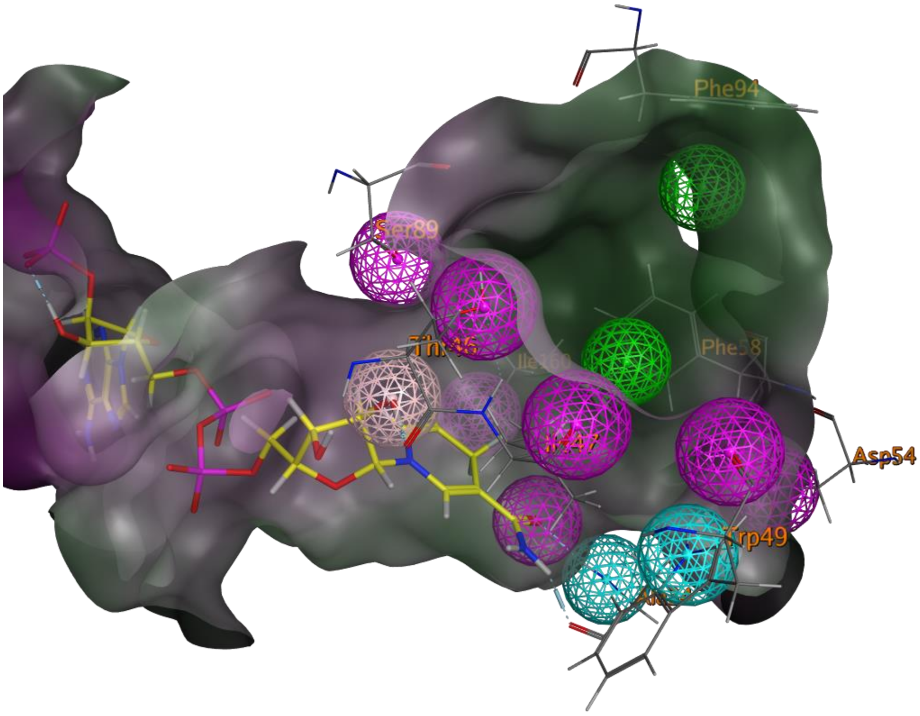

Figure S15. Target-based pharmacophore hypothesis based on the *Tb*DHFR binding pocket (ID: „3RG9“). Carbon atoms of the co-crystallized co-substrate NADPH in yellow, the molecular surface is coloured according to lipophilicity with lipophilic areas in green and hydrophilic areas in purple. Potential interactions of amino acids with an inhibitor are represented by projecting feature spheres: H-bond donors in purple, H-bond acceptors in cyan, ionic interactions in beige, aromatic centres in orange, hydrophobic structures in green. Exclusion spheres are not depicted.

(A)

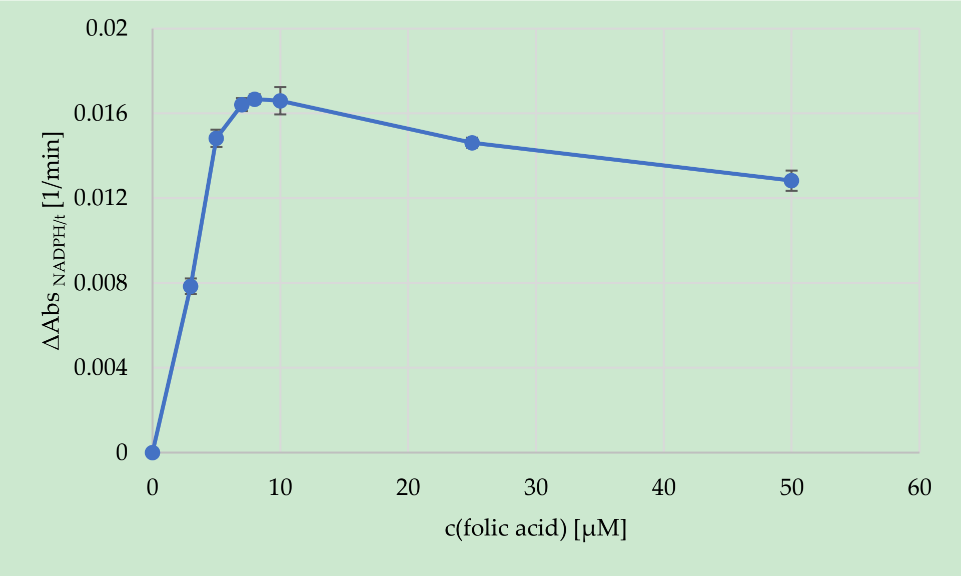

(B)

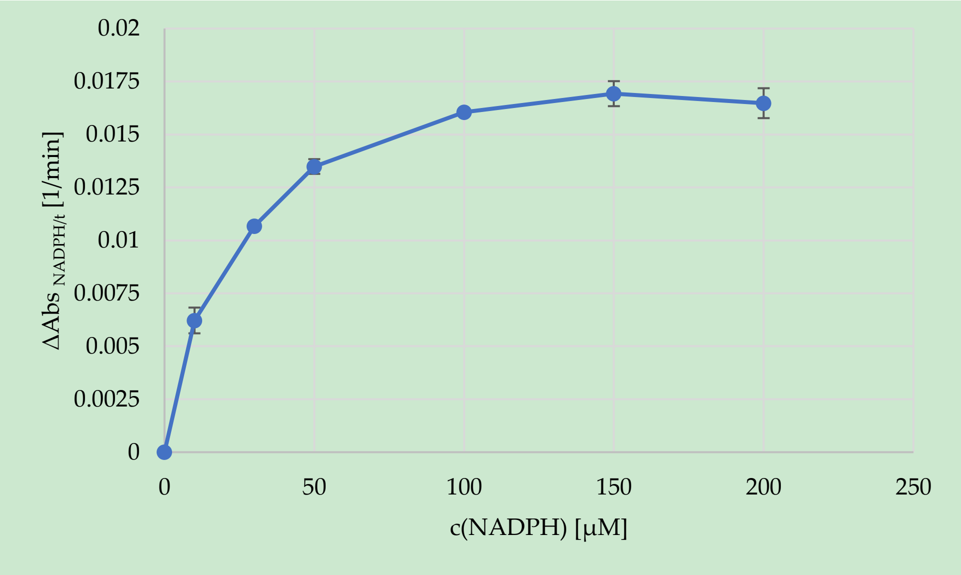

Figure S16. Experimental determination of the saturating conditions of folic acid and NADPH for *Tb*PTR1. (A) Constant concentration of the co-substrate NADPH (200 µM) while varying the concentrations of the substrate folic acid (3 µM – 50 µM). In the concentration range above the saturation (8-10 µM), substrate inhibition was observed. **(B)** Constant concentration of the substrate folic acid (8 µM) while varying the concentrations of the co-substrate NADPH (10 µM – 200 µM). The determination was carried out according to 4.5., using buffer A (50 mM Tris/HCl (pH 7.6), 250 mM NaCl) at 340 nm and a constant temperature of 30 °C.

(A)

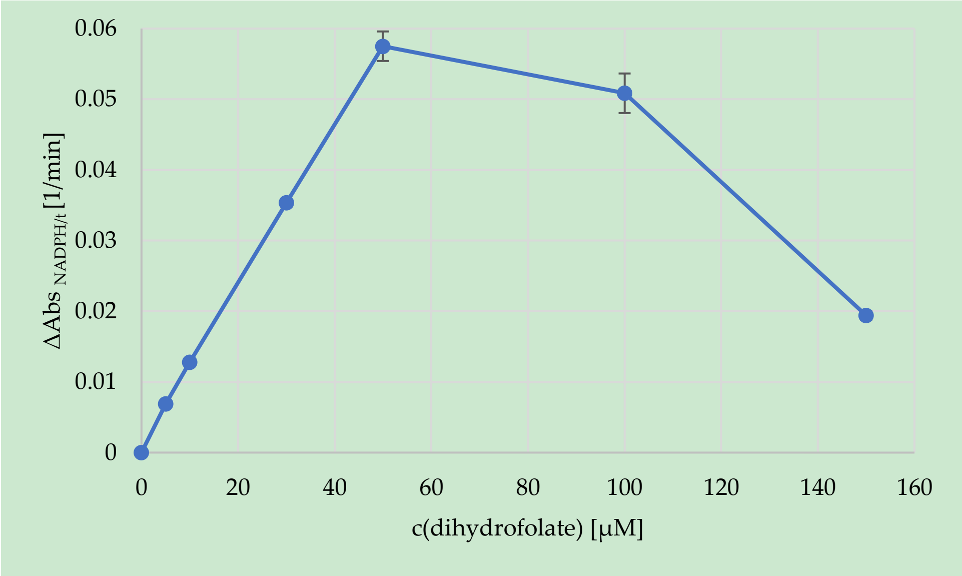

(B)

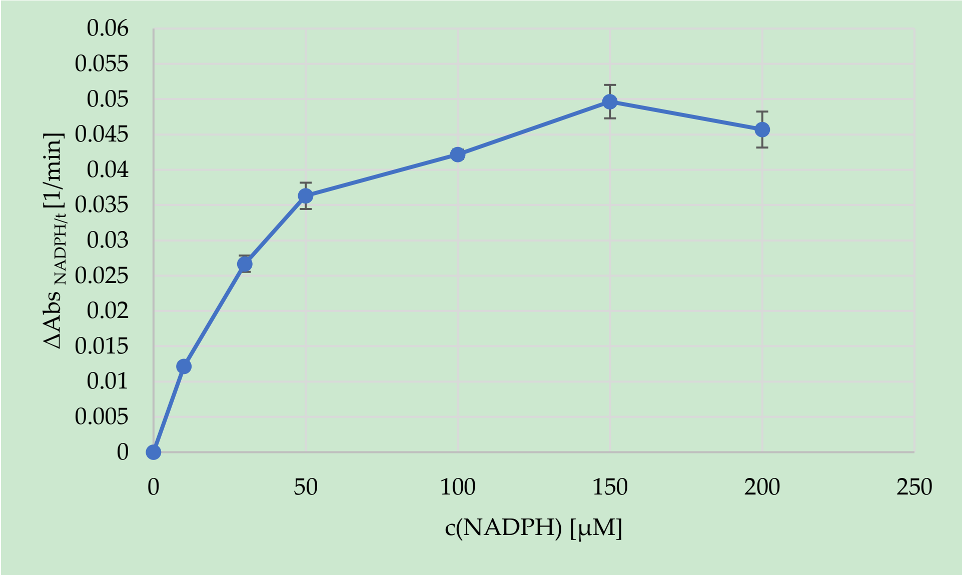

Figure S17. Experimental determination of the saturating conditions of dihydrofolate (DHF) and NADPH for *Tb*DHFR. (**A**) Constant concentration of the co-substrate NADPH (200 µM) while varying the concentrations of the substrate DHF (5 µM – 150 µM). In the concentration range above saturation (>40 µM), substrate inhibition was observed. (**B**) Constant concentration of the substrate DHF (50 µM) while varying the concentrations of the co-substrate NADPH (10 µM – 200 µM). The determination was carried out according to 4.6., using buffer B (50 mM Tris/HCl (pH 7.6), 250 mM NaCl, 10 mM BME) at 340 nm and a constant temperature of 30 °C.

Table S1. Top hits (compound numbers according to Figure 2 and Figure S1) obtained after complex- and target-based pharmacophore search and docking based on protein structure model 2X9G and its co-crystallized inhibitor pemetrexed. Hits are ranked by their docking scores after induced fit docking.

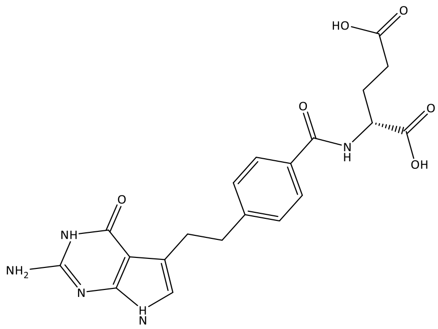

pemetrexed (2X9G)
-9.79 kcal/mol

complex-based		target-based	
**compound**	S	**compound**	S
**8**	-8.16 kcal/mol	**10**	-8.90 kcal/mol
**10**	-8.05 kcal/mol	**9**	-8.21 kcal/mol
**9**	-7.85 kcal/mol	**14**	-7.65 kcal/mol
**14**	-7.55 kcal/mol	**13**	-7.48 kcal/mol
**12**	-7.37 kcal/mol	**15**	-7.27 kcal/mol

Table S2. Top hits (compound numbers according to Figure 2 and Figure S1) obtained after complex- and target-based pharmacophore search and docking based on protein structure model 3MCV and its co-crystallized inhibitor PY848. Hits are ranked by their docking scores after induced fit docking.

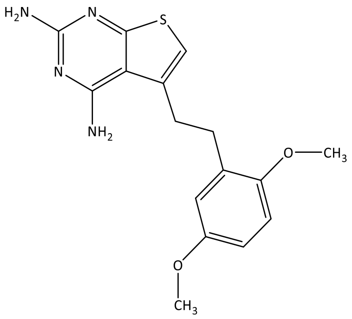

PY848 (3MCV)
-10.37 kcal/mol

complex-based		target-based	
**compound**	S	**compound**	S
**5**	-8.59 kcal/mol	**27**	-7.84 kcal/mol
**4**	-8.03 kcal/mol	**14**	-7.76 kcal/mol
**16**	-7.91 kcal/mol	**1**	-7.15 kcal/mol
**10**	-7.78 kcal/mol	**17**	-7.14 kcal/mol
**14**	-7.76 kcal/mol	**31**	-7.05 kcal/mol

Table S3. Top hits (compound numbers according to Figure 2 and Figure S1) obtained after complex- and target-based pharmacophore search and docking based on protein structure model 4CMJ and its co-crystallized inhibitor 6-(4-bromophenyl)-5-phenyl-7H-pyrrolo[2,3 d]pyrimidine-2,4-diamine. Hits are ranked by their docking scores after induced fit docking.

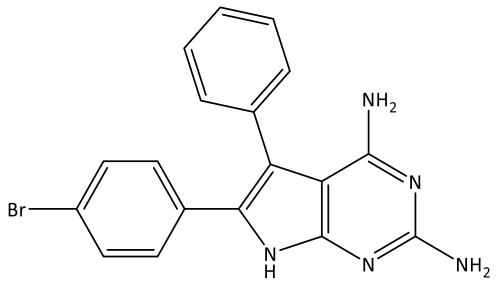

6-(4 bromophenyl)-5-phenyl-7H-pyrrolo[2,3 d]pyrimidine-2,4-diamine (4CMJ)
-9.51 kcal/mol

complex-based		target-based	
**compound**	S	**compound**	S
**10**	-8.90 kcal/mol	**9**	-9.09 kcal/mol
**31**	-8.88 kcal/mol	**1**	-8.81 kcal/mol
**19**	-8.49 kcal/mol	**31**	-8.52 kcal/mol
**7**	-8.31 kcal/mol	**19**	-8.31 kcal/mol
**3**	-8.27 kcal/mol	**20**	-8.23 kcal/mol

Table S4. Top hits (compound numbers according to Figure 2 and Figure S1) obtained after complex- and target-based pharmacophore search and docking based on protein structure model 4CMK and its co-crystallized inhibitor 2-amino-5-phenethyl-6-phenyl-3H-pyrrolo[2,3-d]pyrimidine-4(7H)-one. Hits are ranked by their docking scores after induced fit docking.

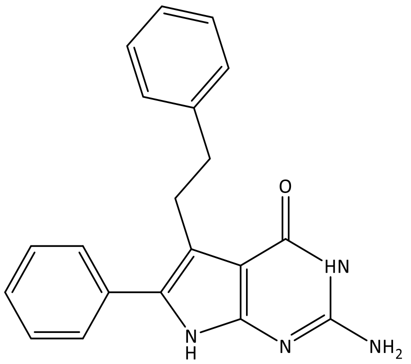

2-amino-5-phenethyl-6-phenyl-3H-pyrrolo[2,3 d]pyrimidine-4(7H)-one (4CMK)
-8.77 kcal/mol

complex-based		target-based	
**compound**	S	**compound**	S
**10**	-9.12 kcal/mol	**11**	-8.63 kcal/mol
**9**	-9.01 kcal/mol	**5**	-8.55 kcal/mol
**21**	-8.83 kcal/mol	**21**	-8.48 kcal/mol
**19**	-8.75 kcal/mol	**7**	-8.32 kcal/mol
**5**	-8.65 kcal/mol	**22**	-8.32 kcal/mol

Table S5. Top hits (compound numbers according to Figure 2 and Figure S1) obtained after complex- and target-based pharmacophore search and docking based on protein structure model 5JDI and its co-crystallized inhibitor 3,6-dihydroxy-2-(3-hydroxyphenyl)-4H-1-benzopyran-4-one. Hits are ranked by their docking scores after induced fit docking.

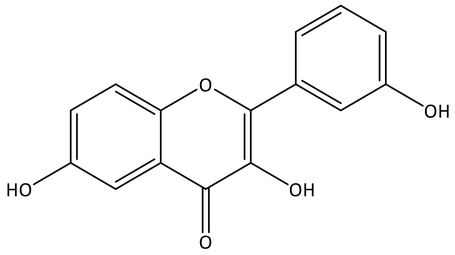

3,6-dihydroxy-2-(3-hydroxyphenyl)-4H-1-benzopyran-4-one (5JDI)
-7.65 kcal/mol

complex-based		target-based	
**compound**	S	**compound**	S
**3**	-8.95 kcal/mol	**7**	-9.39 kcal/mol
**23**	-8.73 kcal/mol	**11**	-8.99 kcal/mol
**19**	-8.65 kcal/mol	**25**	-8.56 kcal/mol
**26**	-8.60 kcal/mol	**16**	-8.53 kcal/mol
**22**	-8.50 kcal/mol	**31**	-8.33 kcal/mol

Table S6. Top hits (compound numbers according to Figure 2 and Figure S1) obtained after complex- and target-based pharmacophore search and docking based on protein structure model 3QFX and its co-crystallized inhibitor pyrimethamine. Hits are ranked by their docking scores after induced fit docking.

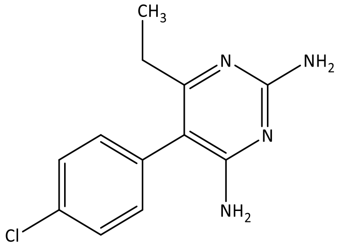

pyrimethamine (3QFX)
-8.26 kcal/mol

complex-based		target-based	
**compound**	S	**compound**	S
**5**	-8.81 kcal/mol	**4**	-8.44 kcal/mol
**8**	-8.42 kcal/mol	**24**	-8.07 kcal/mol
**4**	-8.32 kcal/mol	**27**	-7.83 kcal/mol
**10**	-8.32 kcal/mol	**28**	-7.81 kcal/mol
**24**	-8.09 kcal/mol	**29**	-7.79 kcal/mol

Table S7. Top hits (compound numbers according to Figure 2 and Figure S1) obtained after complex- and target-based pharmacophore search and docking based on protein structure model 3RG9 and its co-crystallized inhibitor WR99210. Hits are ranked by their docking scores after induced fit docking.

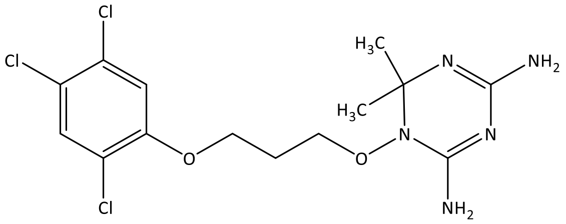

WR99210 (3RG9)
-8.66 kcal/mol

complex-based		target-based	
**compound**	**S**	**compound**	**S**
**9**	-9.63 kcal/mol	**18**	-8.46 kcal/mol
**10**	-8.81 kcal/mol	**31**	-8.18 kcal/mol
**21**	-8.72 kcal/mol	**30**	-8.11 kcal/mol
**8**	-8.55 kcal/mol	**6**	-8.09 kcal/mol
**31**	-8.21 kcal/mol	**27**	-8.00 kcal/mol

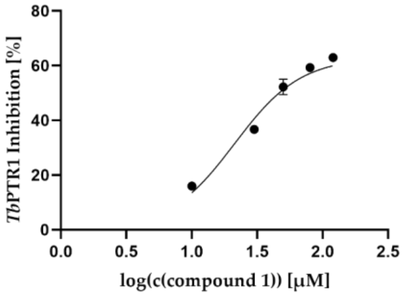

**Best-fit values**	
Bottom	= 0.000
Top	= 62.99
LogEC50	1.327
HillSlope	1.721
EC50	21.24
Span	= 62.99
**95% CI (profile likelihood)**	
LogEC50	1.273 to 1.378
HillSlope	1.474 to 2.020
EC50	18.76 to 23.90
**Goodness of Fit**	
Degrees of Freedom	13
R squared	0.9716
Sum of Squares	127.5
Sy.x	3.132
**Constraints**	
Bottom	Bottom = 0
Top	Top = 62.986
**Number of points**	
# of X values	15
# Y values analyzed	15

Figure S18. Determination of the EC_{50} value of compound **1** against *Tb*PTR1. The EC_{50} value was determined by nonlinear regression analysis using the software GraphPad Prism 8 (Table 1).

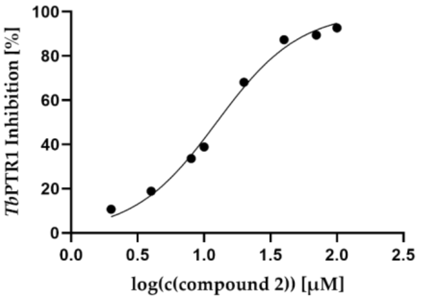

**Best-fit values**	
Bottom	= 0.000
Top	= 100.0
LogEC50	1.095
HillSlope	1.390
EC50	12.44
Span	= 100.0
**95% CI (profile likelihood)**	
LogEC50	1.071 to 1.119
HillSlope	1.288 to 1.504
EC50	11.78 to 13.14
**Goodness of Fit**	
Degrees of Freedom	22
R squared	0.9919
Sum of Squares	191.1
Sy.x	2.947
**Constraints**	
Bottom	Bottom = 0
Top	Top = 100
**Number of points**	
# of X values	24
# Y values analyzed	24

Figure S19. Determination of the IC_{50} value of compound **2** against *Tb*PTR1. The IC_{50} value was determined by nonlinear regression analysis using the software GraphPad Prism 8 (Table 1).

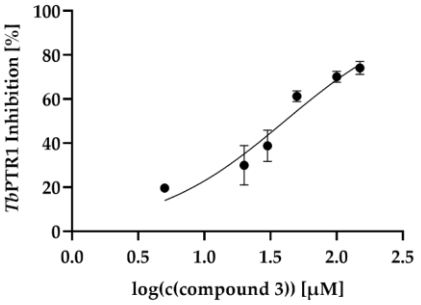

**Best-fit values**	
Bottom	= 0.000
Top	= 100.0
LogEC50	1.607
HillSlope	0.8690
EC50	40.45
Span	= 100.0
**95% CI (profile likelihood)**	
LogEC50	1.527 to 1.685
HillSlope	0.6881 to 1.084
EC50	33.62 to 48.39
**Goodness of Fit**	
Degrees of Freedom	16
R squared	0.9126
Sum of Squares	697.6
Sy.x	6.603
**Constraints**	
Bottom	Bottom = 0
Top	Top = 100
**Number of points**	
# of X values	18
# Y values analyzed	18

Figure S20. Determination of the IC_{50} value of compound **3** for *Tb*PTR1. The IC_{50} value was determined by nonlinear regression analysis using the software GraphPad Prism 8 (Table 1).

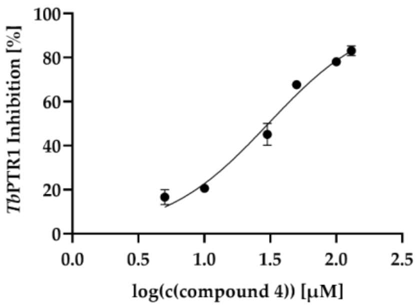

**Best-fit values**	
Bottom	= 0.000
Top	= 100.0
LogEC50	1.485
HillSlope	1.096
EC50	30.54
Span	= 100.0
**95% CI (profile likelihood)**	
LogEC50	1.438 to 1.531
HillSlope	0.9707 to 1.238
EC50	27.41 to 33.93
**Goodness of Fit**	
Degrees of Freedom	16
R squared	0.9771
Sum of Squares	289.8
Sy.x	4.256
**Constraints**	
Bottom	Bottom = 0
Top	Top = 100
**Number of points**	
# of X values	18
# Y values analyzed	18

Figure S21. Determination of the IC_{50} value of compound **4** for *Tb*PTR1. The IC_{50} value was determined by nonlinear regression analysis using the software GraphPad Prism 8 (Table 1).

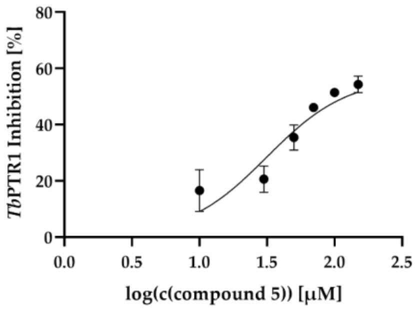

**Best-fit values**	
Bottom	= 0.000
Top	= 56.92
LogEC50	1.499
HillSlope	1.452
EC50	31.52
Span	= 56.92
**95% CI (profile likelihood)**	
LogEC50	1.367 to 1.606
HillSlope	1.008 to 2.236
EC50	23.27 to 40.33
**Goodness of Fit**	
Degrees of Freedom	16
R squared	0.8468
Sum of Squares	620.5
Sy.x	6.227
**Constraints**	
Bottom	Bottom = 0
Top	Top = 56.92
**Number of points**	
# of X values	18
# Y values analyzed	18

Figure S22. Determination of the EC_{50} value of compound **5** for *Tb*PTR1. The EC_{50} value was determined by nonlinear regression analysis using the software GraphPad Prism 8 (Table 1).

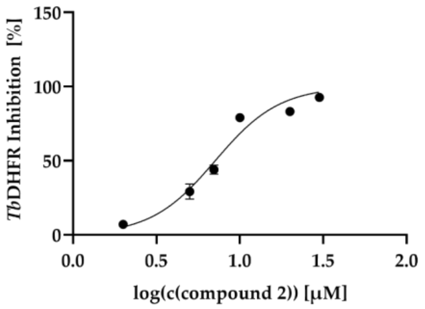

**Best-fit values**	
Bottom	= 0.000
Top	= 100.0
LogEC50	0.8527
HillSlope	2.259
EC50	7.124
Span	= 100.0
**95% CI (profile likelihood)**	
LogEC50	0.8122 to 0.8931
HillSlope	1.733 to 3.120
EC50	6.489 to 7.818
**Goodness of Fit**	
Degrees of Freedom	16
R squared	0.9565
Sum of Squares	771.9
Sy.x	6.946
**Constraints**	
Bottom	Bottom = 0
Top	Top = 100
**Number of points**	
# of X values	18
# Y values analyzed	18

Figure S23. Determination of the IC_{50} value of compound **2** for *Tb*DHFR. The IC_{50} value was determined by nonlinear regression analysis using the software GraphPad Prism 8 (Table 1).

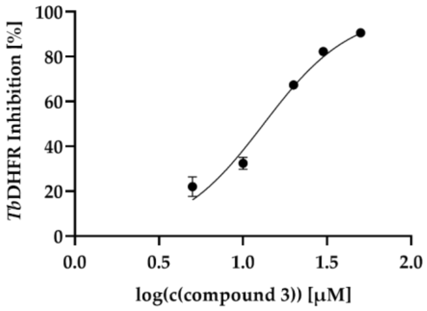

**Best-fit values**	
Bottom	= 0.000
Top	= 100.0
LogEC50	1.123
HillSlope	1.691
EC50	13.27
Span	= 100.0
**95% CI (profile likelihood)**	
LogEC50	1.083 to 1.161
HillSlope	1.457 to 1.964
EC50	12.11 to 14.50
**Goodness of Fit**	
Degrees of Freedom	13
R squared	0.9745
Sum of Squares	283.3
Sy.x	4.668
**Constraints**	
Bottom	Bottom = 0
Top	Top = 100
**Number of points**	
# of X values	15
# Y values analyzed	15

Figure S24. Determination of the IC_{50} value of compound **3** for *Tb*DHFR. The IC_{50} value was determined by nonlinear regression analysis using the software GraphPad Prism 8 (Table 1).
